# Pancreatic enzyme activity in the bile of healthy cats and its association with biliary morphology

**DOI:** 10.1111/jvim.16196

**Published:** 2021-06-12

**Authors:** Shinsuke Fujimoto, Shidow Torisu, Yasuyuki Kaneko, Shinya Mizutani, Shushi Yamamoto, Kiyokazu Naganobu, Kensuke Nakamura

**Affiliations:** ^1^ Miyazaki University Veterinary Medical Teaching Hospital Laboratory 1‐1 Gakuenkibanadainishi Miyazaki City Miyazaki Prefecture Japan; ^2^ Ozu Animal Clinic 317 Ozumachimuro, Kikuchi‐gun Kumamoto Prefecture Japan; ^3^ Laboratory of Companion Animal Surgery, Department of Companion Animal Clinical Sciences, School of Veterinary Medicine, Rakuno Gakuen University, Ebetsu, Hokkaido, Japan.; ^4^ Laboratory of Veterinary Internal Medicine, Department of Veterinary Clinical Sciences Graduate School of Veterinary Medicine, Hokkaido University 18‐9 Kita, Kita Ward, Sapporo Hokkaido Japan

**Keywords:** cholangitis, cystic duct, pancreatic enzyme activity in bile, pancreatobiliary maljunction

## Abstract

**Background:**

In human medicine, congenital maljunction of the common bile duct (CBD) and main pancreatic duct (MPD), or pancreatobiliary maljunction (PBM), is a known cause of cholecystitis.

**Objective:**

Pancreatic enzyme activity in the bile (a diagnostic marker for PBM) of healthy cats was measured to determine normal values and evaluate its relationship with biliary morphology.

**Animals:**

Fifty‐two healthy cats.

**Methods:**

Cross‐sectional study of the biliary tracts of healthy cats during laparoscopic ovariohysterectomy and measurement of pancreatic enzyme activity in bile. The cats were divided into groups A and B based on the ratio of the diameter of the cystic duct (CD) to the CBD. The normal ratio was 3.4. Pancreatic enzyme activity in bile was compared between the groups.

**Results:**

The CBDs were straight in all cases, whereas the CDs were variably tortuous or dilated. Amylase activity in the bile (median, <100 U/L; range, <100‐591 U/L) was lower than in serum in all cases, and group B, which had a CD/CBD ratio >3.4, had significantly higher amylase activity (median, 109 U/L; range, <100‐591 U/L) in the bile than did group A (median, <100 U/L; range, <100‐238 U/L), which had a CD/CBD ratio <3.4 (*P* = .0009).

**Conclusions and Clinical Importance:**

The results suggest that a dilated CD is associated with reflux of pancreatic juice. In the future, it will be necessary to examine the clinical usefulness of these findings by measuring pancreatic enzyme activity in the bile of cats with cholangitis.

**Abbreviations**
ALB: albumin
ALP: alkaline phosphatase
ALT: alanine transaminase
AMY: amylase activity
AST: aspartate transaminase
BUN: blood urea nitrogen
CBD: common bile duct
Cre: creatinine
ERCP: endoscopic retrograde cholangiopancreatography
LIPA: lipase activity
MPD: main pancreatic duct
PBM: pancreatobiliary maljunction
TBA: total bile acid
TBIL: total bilirubin
TCHO: total cholesterol
TP: total protein
γ‐GPT: γ‐glutamyltransferase

## 1 INTRODUCTION

Cats have the anatomical feature of a Y‐shaped junction of the common bile duct (CBD) and main pancreatic duct (MPD), which is reported to make cholangitis and cholangiohepatitis common, even in relatively young cats.^1^, ^2^, ^3^, ^4^ The pathways of the CBD and MPD in cats and humans are similar.^5^, ^6^ In human medicine, the congenital morphological abnormality called pancreatobiliary maljunction (PBM) causes reflux of pancreatic juice into the biliary tract.^7^, ^8^ In this congenital disease, the CBD and MPD join outside the duodenal wall, where the sphincters do not function properly. Pancreatic juice refluxes into the biliary tract and damages the bile duct epithelium, which is thought to cause cholangitis, choleliths, and bile duct cancer.^9^, ^10^, ^11^ Pancreatobiliary majunction is reported to cause congenital biliary dilatation.^7^, ^12^ Congenital biliary dilatation has been referred to as a choledochal cyst.^13^ In human medicine, a definitive diagnosis of PBM usually is made using diagnostic imaging such as endoscopic retrograde cholangiopancreatography (ERCP).^7^, ^8^, ^14^ Also in human medicine, abnormally high pancreatic enzyme activity in the bile has been used as an auxiliary diagnostic test for PBM.^7^, ^9^, ^12^

In veterinary medicine, reports of biliary malformations in cats accompanied by cholangitis include choledochal cyst with congenital dilatation of the biliary tract.^15^, ^16^, ^17^ Pancreatobiliary maljunction was suspected to be involved in these reports. Besides the Y‐shaped junction of the CBD and MPD, which is a normal feature peculiar to cats, biliary malformations such as PBM are thought to cause cholangitis, a common condition in cats. A single report exists in veterinary medicine in which a pediatric endoscope was used to perform ERCP on 4 healthy cats.^18^ The manipulations were difficult, and although the MPD and CBD could be identified simultaneously in 2 cats, in the other 2, only 1 was identified successfully at each scan.^18^ Therefore, using ERCP for the definitive diagnosis of PBM in cats appears to be difficult. Furthermore, because there are no reported reference values for pancreatic enzyme activity in bile in cats, investigating the relationship of PBM in cats with biliary dilatation is difficult.

Therefore, we used laparoscopy to collect bile and investigate biliary system morphology in cats, a species thought to be prone to biliary malformations,^19^ and to measure pancreatic enzyme activity in bile, which has not yet been reported in cats. Furthermore, we examined the relationship between the pancreatic enzyme activity in the bile and biliary morphology.

## 2 MATERIALS AND METHODS

### 2.1 Animals

Cats that visited the Miyazaki University Veterinary Medical Teaching Hospital, or Ozu Animal Clinic, for laparoscopic ovariohysterectomy between April 2017 and August 2020 were included in the study after their owners gave consent. This study was conducted in compliance with the University of Miyazaki's rules on animal experiments and with the approval of the University's Animal Experiment Ethics Committee (approval no. 2019‐11‐27‐Z23).

### 2.2 Methods

We used a cross‐sectional design in this study. Serum biochemistry, thoracic radiography, and abdominal ultrasonography were performed before laparoscopic ovariohysterectomy. Thoracic radiography and abdominal ultrasonography confirmed that no obvious abnormalities were present before surgery in all cases. Serum biochemistry was performed using a dry chemistry analyzer in the hospital (Catalyst One, IDEXX, Tokyo, Japan). Serum biochemistry tests measured concentrations or activities of total protein (TP), albumin (ALB), alanine transaminase (ALT), aspartate transaminase (AST), alkaline phosphatase (ALP), γ‐glutamyltransferase (γ‐GPT), total cholesterol (TCHO), total bilirubin (TBIL), blood urea nitrogen (BUN), and creatinine (Cre). Total bile acid (TBA) concentration was measured at the same time using another dry chemical analyzer (FUJI DRI‐CHEM IMMUNE AU10, Fujifilm Corp, Tokyo, Japan). Amylase (AMY) and lipase (LIPA) activities were measured by Fujifilm Corporation (Fukuoka, Japan).

### 2.3 Observation of the biliary system

**FIGURE 1:**
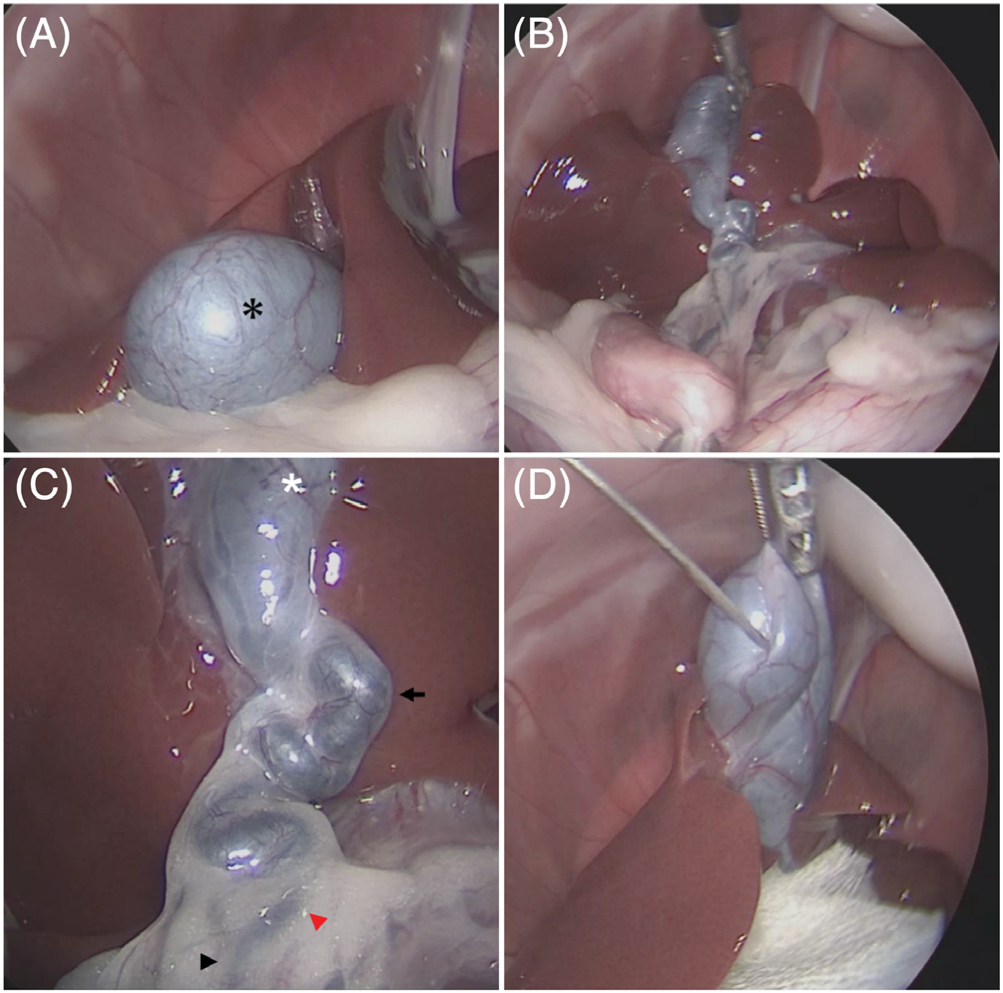
Laparoscopic observation of the biliary system was performed as follows. First, the gallbladder was observed under endoscopy from the fundus to the gallbladder body (A). As shown, the gallbladder (*) can be observed with a laparoscope. Afterward, by grasping and pulling the gallbladder and duodenum with laparoscopic Babcock forceps, it was possible to visually examine the entire image of the liver, gallbladder, cystic duct, and common bile duct (B). Further, the gallbladder (*), cystic duct (arrow), common bile duct (arrowhead), and hepatic duct (red arrowhead) can be observed in detail when approached with a rigid scope (C). Then, laparoscopic cholecystocentesis was performed to collect a bile sample. Under laparoscopy, the puncture could be performed while directly observing the gallbladder. Gauze (*) was used to protect the organs to ensure that the bile sample could be collected safely (D)

Cats were fasted for at least 12 hours and sedated using a mixture of midazolam at a dosage of 0.2 mg/kg and butorphanol at a dosage of 0.2 mg/kg administered IV. Propofol at a dosage of 0.5 mg/kg was administered IV to effect, followed by tracheal intubation to maintain anesthesia using 100% O_2_ and isoflurane inhalation. After laparoscopic ovariohysterectomy, we recorded the morphology from the gallbladder to the CBD. Then, a needle (23 G, 1^1^/_4_ inch) was inserted aseptically into the gallbladder to collect bile (Figure 1). The color of the collected bile was recorded, the volume and AMY and LIPA activity of the bile were measured, and aerobic and anaerobic bacterial culture and identification were performed at the same time.

### 2.4 Measurement of pancreatic enzyme activity in bile

To measure the pancreatic enzyme activity in bile, we used the same serum‐based method as used in the human medical field. In humans, AMY is measured when the pancreatic enzyme activity in the bile is used as an auxiliary diagnostic test for PBM.^7^, ^14^ In our study, serum and bile AMY were measured using the JSCC transferable method, an enzymatic method using 4,6‐ethylidene‐(G7)‐p‐nitrophenyl‐(G1)‐α:d‐maltohepatoside, similar to that used in human medicine. However, the lower limit of detection using this method is 100 U/L. Thus, for statistical purposes, results <100 U/L were recorded as 50 U/L. Serum and bile LIPA were measured at the same time. Lipase was measured using the DGGR substrate method, which has high specificity for the detection of feline pancreatic lipase activity.^20^ Finally, AMY and LIPA in bile and serum were compared based on measurement results.

### 2.5 Morphological analysis of the gallbladder, cystic duct, and CBD

We performed detailed macroscopic observations of the morphology of the biliary system (gallbladder, cystic duct [CD], CBD) using endoscopy to evaluate for the presence of biliary malformations. Laparoscopy was performed using an endoscopic camera (Veterinary Video Camera III, Karl Storz Veterinary Endoscopy), light source (Xenon nova, Karl Storz Veterinary Endoscopy), and a 5‐mm Hopkins II forward‐oblique telescope 0° (Karl Storz Veterinary Endoscopy).

Gallbladder malformations were classified based on previous reports in cats, as double, bilobed, or trilobed gallbladder.^19^, ^21^, ^22^

**FIGURE 2:**
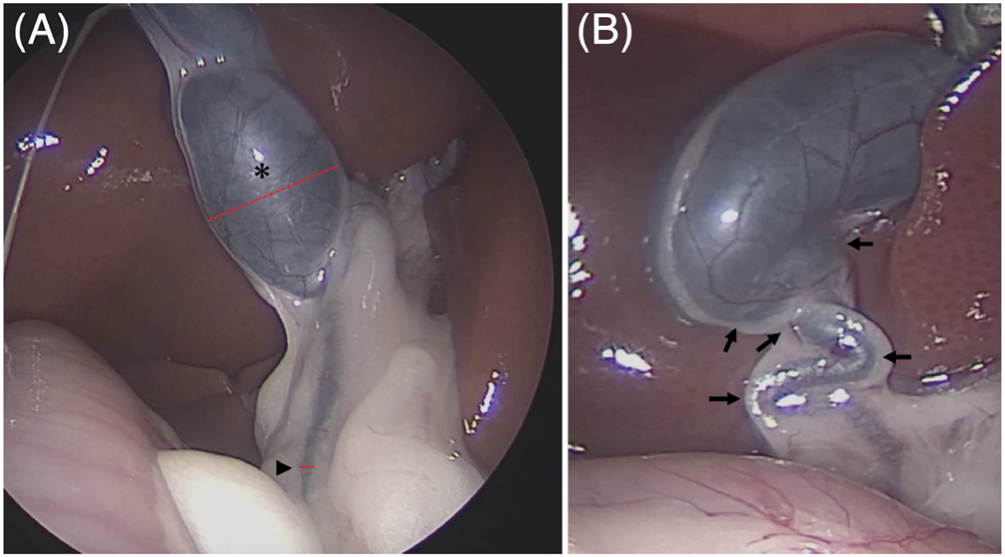
Dilatation of the cystic duct (CD) was measured as follows. With the CD and common bile duct (CBD) visible simultaneously in a single endoscopic image, we calculated the ratio between the width of the thickest part (dotted line) of the CD (*) and the width of the thinnest part (solid line) of the CBD (arrowhead; CD/CBD). For example, in the image of the biliary system of the case in (A), the width of the CD using the scale on the image is 2.94 and the width of the common bile duct is 0.32, implying that the dilatation is 9.18‐fold. In addition, the number of tortuous turns was obtained by adding the total number of places where the biliary tract from the CD to the common bile duct tortuous turns by ≥90° (arrow). Therefore, the total number of tortuous turns in the case in (B) is 5

Tortuosity of the CD or CBD is commonly observed in healthy cats,^21^, ^22^, ^23^ and clearly identifying the transition between the CD and the CBD in cats has been difficult.^21^, ^22^, ^23^ In both the medical and veterinary fields, the CD is defined as the conduit that connects the gallbladder to the CBD, and the CBD as the conduit from the junction of the left and right hepatic ducts to the duodenum.^23^ We measured the diameter of the CD and CBD based on this definition. With the CD and CBD visible simultaneously in a single endoscopic image, we calculated the ratio between the width of the thickest part of the CD and the width of the thinnest part of the CBD (CD/CBD; Figure 2A). The median CD/CBD of all cats was calculated, and the cats were separated into a group with values lower than the median (group A) and a group with values higher than the median (group B). Furthermore, regardless of the severity of dilatation, tortuosity of the biliary tract was observed to a variable extent in almost all cats. Therefore, each tortuous turn of ≥90° in the biliary tract from the CD to the CBD was counted to obtain a total number of tortuous turns for each cat (Figure 2B). The frequency of gallbladder malformations, the number of tortuous turns, and pancreatic enzyme activity in serum and bile were compared between groups.

### 2.6 Statistical analysis

The program JMP13 (SAS Institute Inc, Cary, North Carolina) was used for statistical analysis. All data are expressed as median (range, minimum‐maximum) and were evaluated using a nonparametric test. The Wilcoxon test was used to compare the 2 groups. *P* < .05 was considered statistically significant.

## 3 RESULTS

### 3.1 Subject animals

The subjects were 60 cats, with a median age of 10 months (range, 5‐144 months), that were judged to be in good health, with a normal appetite and no obvious clinical signs, based on interviews with the owners and physical examinations conducted during the study. Six cats were excluded because of slightly increased liver enzyme activity serum biochemistry test results. Bile samples were collected and tested in the remaining 54 cats. Cholecystocentesis was performed to collect bile samples during laparoscopy. Vomiting and loss of appetite were observed in 3 cats the day after laparoscopy, but no obvious leakage of bile into the abdominal cavity occurred, and the clinical signs improved the next day with symptomatic treatment such as IV fluids and antiemetics. Therefore, no serious complications were observed with the procedure. The amount of bile collected was 0.3‐4.5 mL in volume (median, 1.0 mL) and <2.0 mL in 43 of the 54 cats (79.6%). The bile was dark yellow in 28 cats, dark green in 25 cats, and light brown in 1 cat. The largest bile sample was light brown and 4.5 mL in volume. Bile culture results were negative in 52 cats and positive in 2 cats. In the positive cases, *Micrococcus luteus* and *Corynebacterium* sp. were identified in the bile. A previous study^24^ reported that bacteria were found in the bile in approximately 4% of normal cats, and although the serum biochemistry results in these 2 cats showed no obvious abnormalities, the presence of bacteria in the bile may have affected the morphology of the biliary system and measurements of AMY and LIPA in bile. Therefore, these 2 cats were excluded, and the remaining 52 cats were evaluated (Table 1).

**TABLE 1:** Measurements of each test item for groups classified based on the degree of cystic duct dilatation of the 52 cases included in this study

	Group A (range: min‐max)	Group B (range: min‐max)	Reference range	*P*‐value
Number	27	25	N/A	N/A
Age (months)	11 (5–144)	8 (6‐12)	N/A	.03
BW (kg)	3.3 (2.3‐4.6)	2.9 (2.3‐4.1)	N/A	.03
Variety	Mixed 26, Bengal 1	Mixed 24, Munchkin 1	N/A	1
CD/CBD (factor of dilatation)	2.5 (1.5‐3.4)	5 (3.6‐10)	N/A	<.0001
Number of tortuous turns	4 (2–8)	3 (3–7)	N/A	.35
GB malformation (y/n)	4/27	3/25	N/A	1
TP (g/dL)	6.9 (6.0‐8.4)	7.1 (6.2‐8.9)	5.7‐8.9	.24
ALB (g/dL)	3.3 (2.7‐3.9)	3.2 (2.9‐3.7)	2.2‐4.0	.99
ALT (U/L)	66 (41‐84)	73 (43‐84)	22‐84	.33
AST (U/L)	12 (6‐25)	15 (8‐22)	18‐51	.14
ALP (U/L)	72 (25‐112)	98 (50‐111)	0‐165	.29
γ‐GPT (U/L)	2 (1–8)	2 (1–7)	0‐10	.28
TBIL (mg/dL)	0.1 (0.1‐0.3)	0.1 (0.1–0.3)	<0.4	.2
TCHO (mg/dL)	94 (66‐181)	99 (69‐172)	65‐225	.96
TBA (μmol/L)	2.2 (1.0‐4.5)	2.0 (1.0‐3.1)	<5	.43
BUN (mg/dL)	23 (16‐30)	23 (14‐30)	16‐36	.19
Cre (mg/dL)	1.2 (0.5‐1.8)	1.2 (0.8‐1.8)	0.8‐2.4	.61
AMY (U/L)	1068 (503‐2033)	1332 (672‐2123)	607‐2846	.07
LIPA (U/L)	20 (6‐22)	20 (5‐25)	<30	.2
Bile AMY (U/L)	50^a^ (50^a^–238)	109 (50^a^–591)	N/A	.0009
Bile LIPA (U/L)	29 (19‐124)	30 (18–754)	N/A	.44
Bile AMY/AMY	0.056 (0.024‐0.136)	0.082 (0.027‐0.428)	N/A	.02

Abbreviations: ALB, albumin; ALP, alkaline phosphatase; ALT, alanine transaminase; AMY, amylase activity; AST, aspartate transaminase; BUN, blood urea nitrogen; CBD, common bile duct; CD, cystic duct; Cre, creatinine; LIPA, lipase activity; N/A, not applicable; TBA, total bile acid; TBIL, total bilirubin; TCHO, total cholesterol; TP, total protein; γ‐GPT, γ‐glutamyltransferase.

^a^ Bile AMY ≤100 U/L was considered 50.

### 3.2 Laparoscopic morphological findings of the biliary system

**FIGURE 3:**
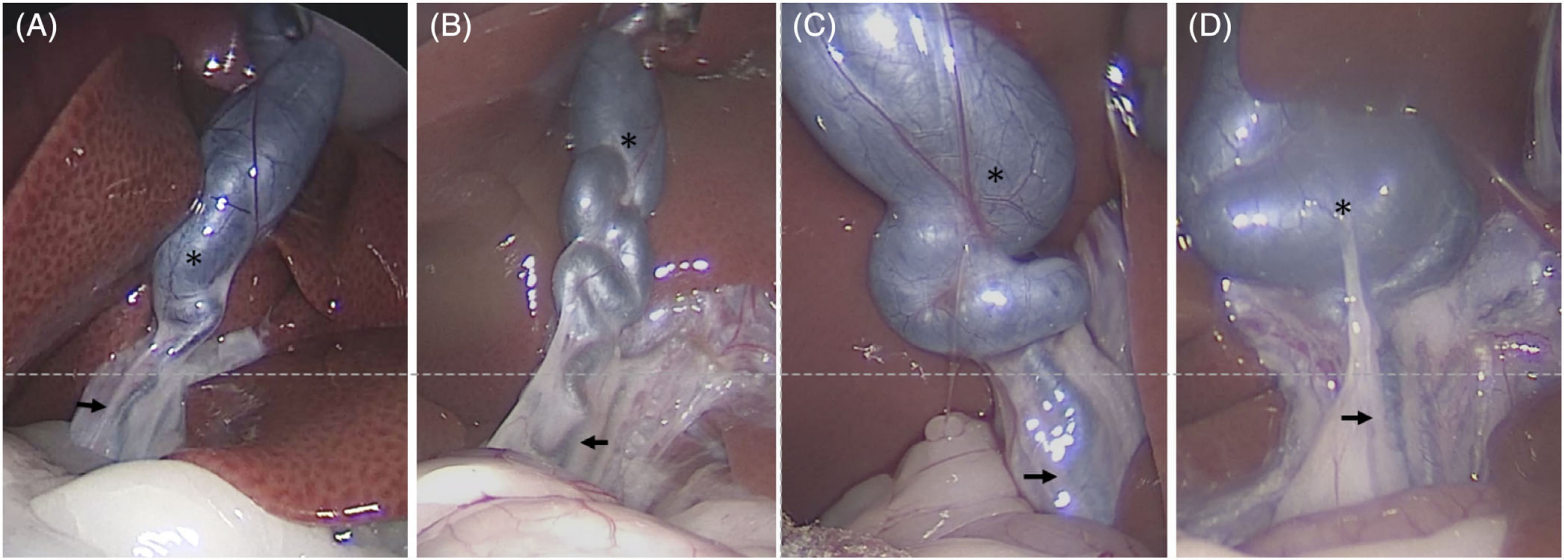
Biliary morphology was classified based on the degree of cystic duct dilatation. Cases in which the minor axis of the cystic duct (*) was ≤3.4 times the minor axis of the common bile duct (arrow) were placed in the mild dilatation group and those in which the minor axis of the cystic duct (*) was ≥3.5 times the minor axis of the common bile duct (arrow) were placed in the severe dilatation group. The mild dilatation group (A, B) had 27 of the 52 cases, whereas the severe dilatation group (C, D) had 25 cases. The photographs show 2 examples from each group

Laparoscopy indicated that the main site of tortuosity was the CD. Furthermore, the junction of the CBD and the hepatic duct was not visually distinct because of fat adhering to the biliary tract. The CBDs that could be confirmed by laparoscopy were nearly straight. In contrast, several different morphologies were observed for the CD, depending on the extent of dilatation and tortuosity. Moreover, the more severe the dilatation of the CD, the more distinct the morphology (Figure 3). Therefore, differences in the extent of CD dilatation appear to be a factor in the formation of distinctive biliary system morphologies in cats.

**FIGURE 4:**
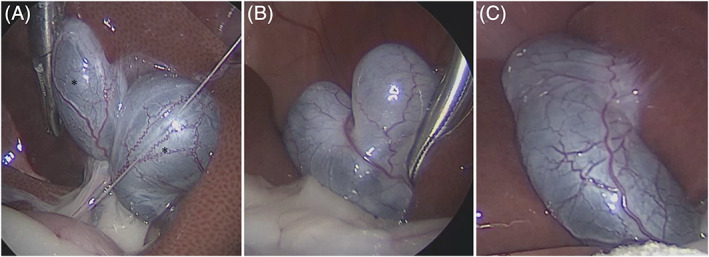
A double gallbladder was observed in 1 of the 52 cats in this study. Gallbladders (*) were found in the quadrate lobe and the medial right lobe, respectively (A). A 2‐lobed (heart‐shaped) gallbladder was observed in 7 of the 52 cats (B). A normal oval‐shaped gallbladder was observed in 44 of the 52 cats (C). The photographs show typical examples of each type

Three types of gallbladder morphology were observed: double gallbladder (1 cat, Figure 4A), bilobed (heart‐shaped) gallbladder (7 cats, Figure 4B), and normal (oval‐shaped) gallbladder (44 cats, Figure 4C). Thus, the frequency of gallbladder malformation in our study was 15.3% (8/52 cats).

The CD/CBD ratio of the 52 cats was 3.4 (range, 1.5‐10). The CD/CBD ratio was ≤3.4 in group A, which consisted of 27 cats (51.9%), and ≥3.5 in group B, which consisted of 25 cats (48.1%).

The median number of tortuous turns in the 52 cats was 3 (range, 2‐8), with the number of tortuous turns not being significantly different between groups A and B (Table 1).

### 3.3 Bile and blood tests

In all 52 cats, bile AMY was lower than serum AMY. Median bile AMY was <100 (range, <100‐591) U/L, with 32 cats (61.5%) having activity below the detection limit (<100 U/L). In contrast, median bile LIPA was 29 (range, 18‐754) U/L, and bile LIPA was higher than serum LIPA in 51 cats (98.1%).

**FIGURE 5:**
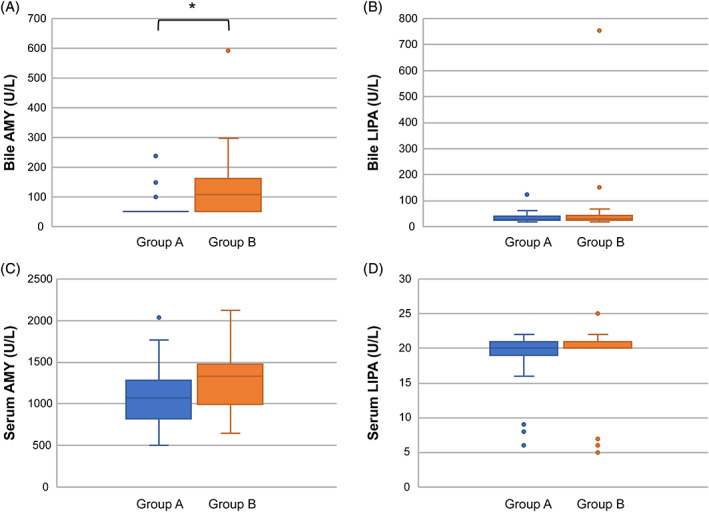
Comparison of AMY and LIPA in the serum and bile between groups A and B. Each value is shown with box‐and‐whisker plots and dots. The black line indicates the median value, and the box indicates the interquartile range (IQR [25th to 75th percentile]). The dots outside the box‐and‐whisker plot either fall below the value obtained by subtracting 1.5 times the interquartile range from the first quartile or exceed the value obtained by adding 1.5 times the quartile range to the third quartile. The dot indicates that there may be an outlier. Furthermore, 22 of the 27 individuals in group A were <100 U/L (below the detection limit), and only the remaining 5 individuals were 100 U/L or higher (100 U/L, *n* = 2; 148 U/L, *n* = 1; 149 U/L, *n* = 1; 238 U/L, *n* = 1); these are represented by dots only. The reason there are only 3 dots is that the dots for the 2 individuals with 100 U/L overlapped, and the 2 individuals with 148 and 149 U/L were almost the same numbers; hence, these dots overlapped. Bile AMY was significantly higher in group B than in group A (*P = .0009). Significant differences were not observed for serum AMY or serum and bile LIPA. AMY, amylase activity; IQR, interquartile range; LIPA, lipase activity

**FIGURE 6:**
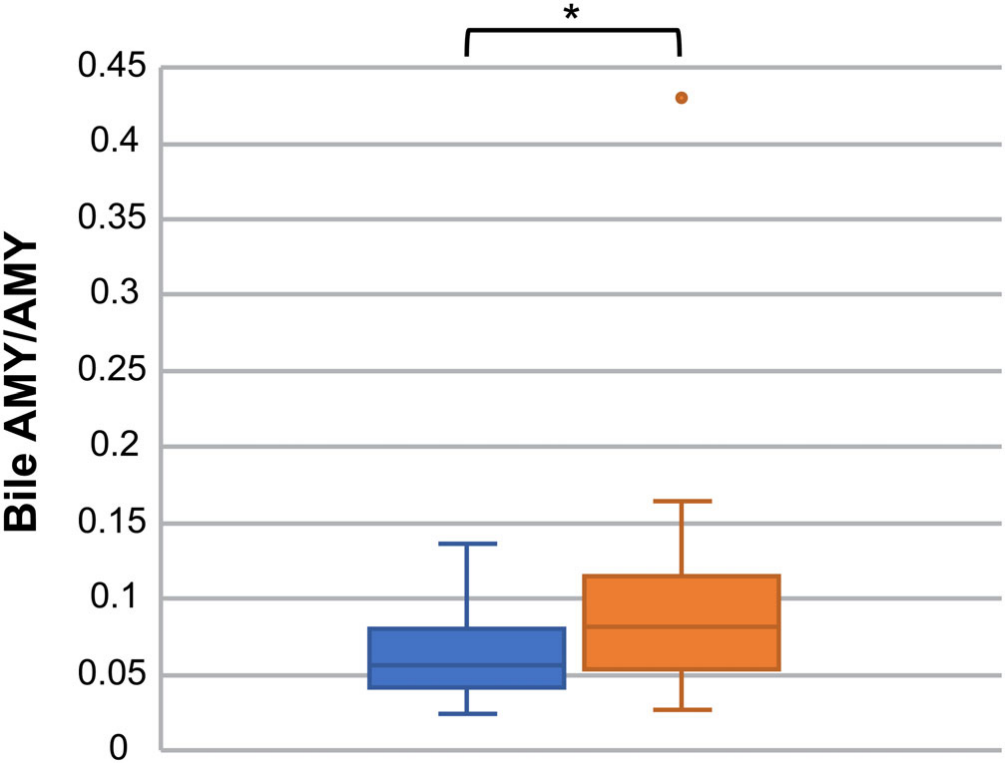
Comparison of bile AMY/AMY between groups A and B. Each value is shown with box‐and‐whisker plots and dots. The black line indicates the median value, and the box indicates the interquartile range (IQR [25th to 75th percentile]). The dots outside the box‐and‐whisker plot either fall below the value obtained by subtracting 1.5 times the interquartile range from the first quartile or exceed the value obtained by adding 1.5 times the quartile range to the third quartile. The dot indicates that there may be an outlier. Moreover, 22 of the 27 individuals in group A were <100 U/L (below the detection limit), and only the remaining 5 cats had bile AMY/AMY of 0.05 or higher (0.06, *n* = 1; 0.09, *n* = 1; 0.12, *n* = 2; 0.14, *n* = 1); thus, these are represented by dots only. The reason there are only 4 dots is that the dots for the 2 individuals with .12 overlapped. The ratio of amylase activity in bile (bile AMY) to amylase activity in serum (AMY) is set as bile AMY/AMY, and these findings were compared between groups A and B. The higher these ratios, the higher the amylase activity in bile, regardless of the amylase activity in serum. Bile AMY/AMY was significantly higher in group B than in group A (*P = .02). Serum AMY was not significantly different between the groups, which indicates that increased bile AMY in group B is *n*ot related to AMY in serum. AMY: amylase activity; IQR, interquartile range

Comparisons of AMY and LIPA in bile and serum between groups A and B showed that bile AMY was significantly higher in group B (*P* = .0009). However, no significant differences were observed in serum AMY or serum and bile LIPA (Figure 5). Furthermore, when the ratio of bile AMY to serum AMY (bile AMY/AMY) was compared between the groups, bile AMY/AMY was significantly higher in group B than in group A (*P* = .02; Table 1; Figure 6).

**FIGURE 7:**
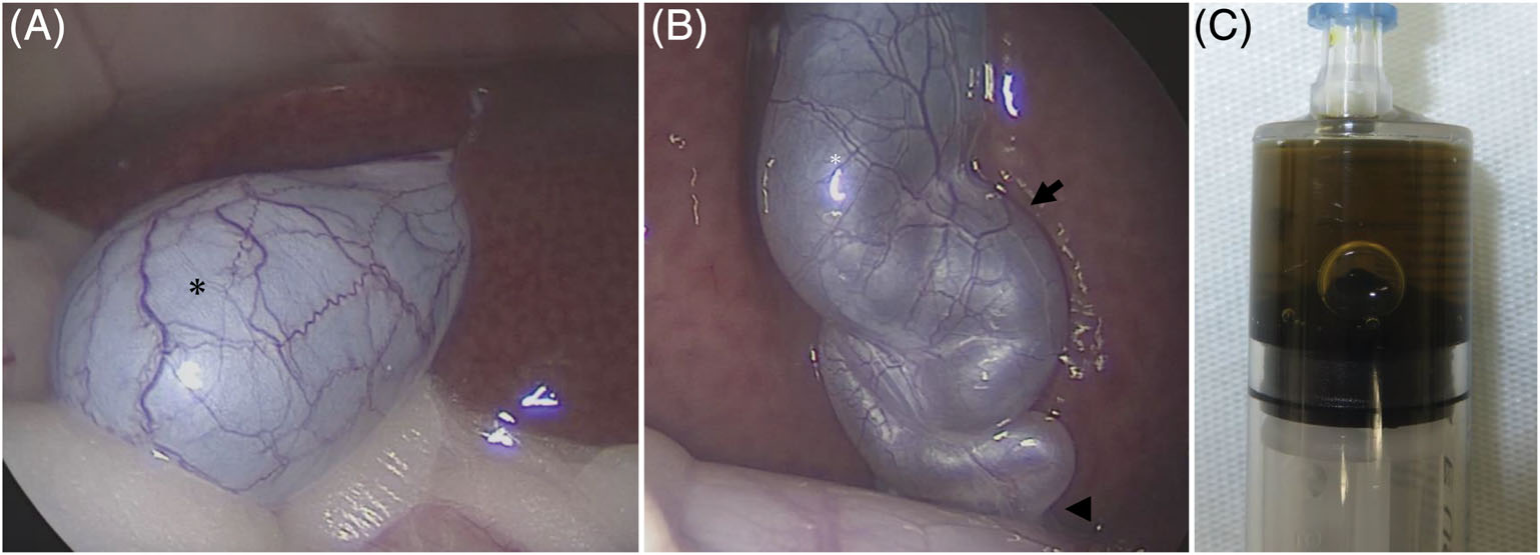
The figure shows the case with the highest activity of bile AMY and LIPA. Observed laparoscopically, the gallbladder had normal morphology (A); however, dilatation and tortuosity were observed throughout the cystic duct (arrow) and common bile duct (arrowhead; B). The bile sample collected by bile puncture was the largest in the study, at 4.5 mL of light‐brown bile (C). AMY, amylase activity; LIPA, lipase activity

In addition, only 1 cat had markedly high bile AMY and LIPA activity (bile AMY, 591 U/L; bile LIPA, 754 U/L; serum AMY, 1378 U/L; serum LIPA, 25 U/L). The bile sample from this cat was light brown in color and 4.5 mL in volume, the largest volume in the study. The CD/CBD ratio in this cat was 7.4 (group B) and uniform dilatation from the CD to the CBD was observed (Figure 7).

## 4 DISCUSSION

Several variations of biliary morphology are reported in cats,^19^, ^22^ but the clinical relevance of these variations is unknown.^21^, ^25^ Additionally, some reports^26^, ^27^, ^28^, ^29^ discuss the relationship between gallstones and cholecystitis, but the details of this relationship are unclear. Therefore, we performed detailed laparoscopic observations of the biliary system morphology in healthy cats that visited our clinic for laparoscopic ovariohysterectomy. The results showed that biliary morphology in healthy cats is characterized by tortuosity and dilatations, with much more variety in morphology compared to dogs. Furthermore, in almost all instances that could be laparoscopically confirmed, the CBD was straight, and tortuosity and dilatations were found mainly in the CD. Biliary tract morphology varied depending on the number of bends and degree of dilatation, but the more severe the dilatation of the CD, the more distinct the morphology. Moreover, the frequency of gallbladder malformation in our study was 15.3% (8/52 cats), which is approximately the same as in previous reports.^19^ The number of cats with gallbladder malformations was approximately the same in groups A and B. Previous reports have suggested an association between gallbladder malformation and hepatobiliary disease,^26^, ^27^, ^28^, ^29^ but we observed no such association.

Our study was the first to measure pancreatic enzyme activity in the bile in cats, which in human medicine is seen as a useful auxiliary diagnostic test for PBM, a cause of congenital biliary dilatation. Choledochal cyst and PBM are thought to be related diseases in cats, but no cases have been definitively diagnosed, and this area remains poorly understood.^16^ Therefore, we measured pancreatic enzyme activity in the bile of healthy cats to investigate the normal activity. As a result, bile AMY was found to be much lower than serum AMY in all cats, but bile LIPA was slightly higher than serum LIPA.

In case of humans, AMY secreted by hepatocytes is said not to be present in the bile of healthy humans.^30^ The source of AMY in bile has been reported to be from the serum via the liver and regurgitation from the pancreatic duct.^31^ Hence, mean bile AMY activity in patients without reflux of pancreatic juice into the bile duct has been reported to be 238 U/L, which is similar to serum AMY activity (130‐400 U/L).^32^ Therefore, increased bile AMY in patients with normal serum AMY is thought to indicate reflux from the pancreatic duct.^33^ However, in our study, in all cats, bile AMY was lower than serum AMY and below the detection limit in 32 cats (61.5%). This finding may be because there is little blood‐derived AMY in the bile of cats or because the JSCC transferable method we used cannot accurately measure bile AMY.

In contrast, bile LIPA was higher than serum LIPA in 51 cats (98.1%). The DGGR method we used to measure LIPA uses 1,2‐o‐dilauryl‐rac‐glycero‐3‐glutaric acid‐(6′‐methyl resorufin)‐ester as the substrate.^34^, ^35^ This substrate is broken down by lipase under alkaline conditions into glutaric acid and methyl resorufin with absorption near 580 nm. This method measures the rate of increase in red absorbance of this methyl resorufin. That is, the substrate used in the DGGR method breaks down faster in bile, which is weakly alkaline, than in serum. Moreover, the optimal pH of lipase is 8, indicating that LIPA would be detected to a higher extent in bile compared to serum.

In addition, in the comparison of pancreatic enzyme activity in the bile between the 2 groups classified by the severity of CD dilatation, bile AMY was markedly higher in group B than in group A, but serum AMY was not significantly different between the groups. As a result, bile AMY/AMY was significantly higher in group B. Thus, increased bile AMY in group B was not associated with serum AMY activity, suggesting reflux of pancreatic juice in the dilated CD.

In human medicine, PBM is a possible cause of reflux of pancreatic juice. In humans, PBM either manifests as congenital biliary dilatation caused by regurgitation of pancreatic juice and inflammatory changes in the inner wall of the bile duct, resulting in cyst‐like dilatation of the bile duct, or as nondilated bile duct type, where good bile duct clearance occurs, with no stagnation of pancreatic juice in the bile duct, and the regurgitated pancreatic juice accumulates in the gallbladder.^7^, ^12^, ^36^ Congenital biliary dilatation is more often symptomatic than is the nondilated bile duct type, and few individuals with nondilated bile duct type are asymptomatic at a young age.^7^ Furthermore, in human medicine, in addition to PBM, secondary papillitis and associated sphincter dysfunction have been reported to be involved in reflux of pancreatic juice in diseases such as bile duct stones, choleliths, and pancreatitis.^37^ The same is conceivable in cats, and in that case, pancreatic juice reflux would simply be a result of the condition. However, the cats used in our study were healthy with no clinical signs and no abnormal laboratory findings, and the cats in our study therefore were very unlikely to have cholangitis. Thus, it is possible that some of the seemingly healthy cats in Group B analyzed in our study could have had biliary malformations that cause reflux of pancreatic juice.

A study of an experimental model of PBM in cats found that the pancreatic enzyme activity in the bile was significantly increased in 9 of the cats in the study compared with 6 normal cats, but no significant change was found in the diameter of the CBD after 6 months.^38^ Therefore, the cats in our study with findings suggestive of regurgitation of pancreatic juice into the biliary tract may develop clinical signs or exhibit dilatation of the CBD at a later time. Furthermore, it is assumed that regurgitated pancreatic juice tends to accumulate in the bent CD in cats because of gravity, and this anatomical feature may cause the CD to dilate without dilatation of the CBD. One cat in group B had extremely high pancreatic enzyme activity in the bile (bile AMY, 591 U/L; bile LIPA, 754 U/L; serum AMY, 1378 U/L; serum LIPA, 25 U/L), and was thought to be a potential PBM case. In human medicine, incidental discovery of pancreatic juice regurgitation into the biliary tract has been reported, even in healthy individuals.^37^ Furthermore, the reference range of bile AMY in humans is ≤1000 U/L^39^ or ≤400 U/L,^40^ which increases to activity as high as 10 000 U/L in PBM,^41^ whereas serum AMY increases to approximately 100 times higher than the reference range. Thus, although the bile AMY of this cat is lower than reports in human medicine, the bile LIPA of this cat was approximately 30 times higher than serum activity, and regurgitation of pancreatic juice into the bile duct was strongly suspected. However, bile LIPA activity is rarely measured for diagnosis of PBM in human medicine, hence the clinical relevance of this measurement currently is unknown. Additional follow‐up investigations are needed to clarify the importance of this finding.

Our study had some limitations. First, the cases were collected from laparoscopic contraceptive surgeries, thus only female cats were studied. However, sex differences in biliary malformations have not been reported previously. Second, we measured pancreatic enzyme activity in the bile using the same method used for serum. Therefore, we cannot rule out possible effects from bile components on the measurement system. However, because the same method is used for humans and is applied clinically, we do not believe this concern is an important problem. Third, there were significant differences in age and weight between the groups (Table 1). In humans, bile AMY tends to increase with age.^37^ If the same is true for cats, age differences could have affected the difference between the groups. Physical confirmation is unlikely to affect bile components, but it could impact the CD and CBD, which may have influenced the grouping of the cats. Fourth, several items were tested statistically, and type 1 error may have occurred. However, the primary endpoints of this study—CD diameter and bile AMY—had sufficiently low *P*‐values, and real differences are likely to have existed between them. Finally, bile AMY was below the detection limit (100 U/L) in several cats, and these cats were evaluated as 50 U/L, which may have affected the results.

## 5 CONCLUSION

We observed tortuosity and dilatation of the CD in young healthy cats. Bile AMY was lower than serum AMY in all cats but was significantly higher in cats with dilated CD. It was hypothesized that reflux of pancreatic juice was associated with a congenital abnormality, such as PBM, and secondary dilatation of the CD. Alternatively, pancreatic juice may regurgitate and accumulate in the gallbladder because of an abnormal CD, but it is assumed that regurgitation would not occur unless some abnormality at the confluence of the pancreatic and bile ducts existed. Nonetheless, our study cannot determine whether these changes will result in cholangitis. The cats included in our study will be followed up, and the frequency of cholangitis between groups A and B compared.

## CONFLICT OF INTEREST DECLARATION

Authors declare no conflict of interest.

## OFF‐LABEL ANTIMICROBIAL DECLARATION

Authors declare no off‐label use of antimicrobials.

## INSTITUTIONAL ANIMAL CARE AND USE COMMITTEE (IACUC) OR OTHER APPROVAL DECLARATION

This study was conducted in compliance with the University of Miyazaki's rules on animal experiments and with the approval of the university's animal experiment ethics committee (approval No.: 2019‐11‐27‐Z23).

## HUMAN ETHICS APPROVAL DECLARATION

Authors declare human ethics approval was not needed for this study.
